# Relationship Between Prenatal Depression and Psychological Capital in Pregnant Women in Guangdong, China: The Mediating Role of Coping Styles

**DOI:** 10.1155/nrp/5368362

**Published:** 2026-05-14

**Authors:** Wenting Liu, Yibing Tan, Weiting Li, Jiagen Xiang, Ming Yang, Min Li, Xiaowan Luo, Yifei Wang, Honglin Chen, Lili Deng

**Affiliations:** ^1^ School of Nursing, Guangzhou University of Chinese Medicine, Guangzhou, Guangdong, China, gzucm.edu.cn; ^2^ Department of Gynecology, Boai Hospital of Zhongshan, Zhongshan, Guangdong, China

**Keywords:** coping style, mediating effect, pregnant women, prenatal depression, psychological capital

## Abstract

**Background:**

Coping styles and psychological capital are both associated with the mental health of pregnant women. However, the relationships among psychological capital, coping styles, and prenatal depression in pregnant women have not yet received sufficient attention. This study examines how coping styles mediate the link between psychological capital and prenatal depression in pregnant women.

**Methods:**

A cross‐sectional study was conducted from March 2024 to December 2024. Pregnant women (*n* = 769) were recruited from two hospitals in Guangdong Province, China. All participants completed the Positive PsyCap Questionnaire, the Simple Coping Style Questionnaire, and the Edinburgh Postnatal Depression Scale. Spearman correlation analysis was applied to explore the relationships among psychological capital, coping style, and prenatal depression and to evaluate the mediating role of coping style.

**Results:**

Prenatal depression was detected in 34.70% of participants. Both psychological capital and positive coping strategies were significantly negatively correlated with prenatal depression (*r* = −0.46, *p* < 0.001 and *r* = −0.26, *p* < 0.001, respectively). In contrast, a significant positive correlation was observed between negative coping strategies and prenatal depression (*r* = 0.18, *p* < 0.001). Psychological capital can directly affect prenatal depression, and coping style is the intermediary variable between psychological capital and depression, with a mediating effect of −0.51 and accounting for approximately 11.87% of the total effect of psychological capital on prenatal depression.

**Conclusion:**

Psychological capital can not only directly affect prenatal depression, but it can also alleviate it by increasing positive coping or suppressing negative coping. Healthcare professionals should pay attention to the mental health status of pregnant women, improve the level of psychological capital, and guide positive responses to improve prenatal depression.

**Summary**
• What does this study contribute to the wider global clinical community?
◦ Psychological capital and its four dimensions (self‐efficacy, optimism, resilience, and hope) were positively correlated with positive coping strategies, enhancing psychological adaptation in pregnant women.
◦ Psychological capital directly reduces prenatal depression risk, serving as a protective factor against depressive symptoms.
◦ Coping strategies mediate the psychological capital–depression link, which explains how psychological resources influence mental health outcomes.
◦ Improving psychological capital (e.g., through resilience training) and modifying coping strategies (e.g., through CBT) offer effective ways to prevent and alleviate prenatal depression.

## 1. Introduction

A systematic review reported that the global average prevalence of prenatal depression in women was 28.50%; however, its prevalence varies widely across countries and according to various patient characteristics [1]. Previous studies have shown that the detection rate of prenatal depression in pregnant Chinese women has reached 24.40%, and 12.80% of pregnant women reported some degree of thoughts of self‐injury [2], thus increasing the risk of emotional problems in offspring [3]. More than 50% of women who experience antenatal depression during pregnancy develop postpartum depression [4]. Therefore, attention to and research on women’s mental health during pregnancy are necessary to better provide pregnant women with mental health management services during pregnancy.

Positive psychological capital, a fundamental construct in positive psychology, represents an individual’s positive psychological state during growth and development and is characterized by four core dimensions: self‐efficacy, optimism, resilience, and hope [5]. Studies have shown that positive psychological capital and its subcomponents are significantly correlated with mental health indicators such as emotional balance, anxiety, and depression [6, 7]. Psychological capital plays a positive role in promoting mental health and is a positive resource for mitigating depression. In previous research, a low level of optimism in psychological capital has been noted to be a risk factor for major depression [8]. The results of a pilot study on positive psychological capital intervention in patients with depression suggest that interventions targeting positive resources may be effective in treating depression, as this is conducive to improving the level of psychological capital in patients with depression and alleviating depressive symptoms [9]. Although there has been research on positive psychology in foreign countries, little is known about this topic in the field of nursing in China. Many studies have focused on adolescents, college students, elderly individuals, medical staff, and other groups [10, 11], but few studies have explored the positive psychological capital of women during pregnancy.

Coping style refers to an individual’s cognitive and behavioral patterns in the face of frustration and stress. Positive coping styles promote an individual’s mental health [12], whereas negative coping styles are often considered predictors of certain negative emotions, such as anxiety and depression [13]. According to the theory of positive psychology, individual psychological problems should be interpreted with a positive attitude, with attention given to the individual’s own positive qualities, positive emotions and experiences, positive individual characteristics, and internal potential, to prevent the emergence of mental health problems and implement effective coping methods. Positive psychological capital can affect individuals’ coping styles, and coping styles play a very important role in mental health regulation [14]. A review of studies on mental health and hope among college students suggested that the level of hope in terms of psychological capital is correlated with an individual’s coping style and is a positive factor in the regulation of depression [15]. Prior research confirms that coping strategies mediate postpartum depression and dual adaptation, highlighting their significant effect on women’s psychological adjustment [16].

Even so, existing research has focused on the relationships between psychological capital and depression [17] or between coping styles and depression [18]. However, few studies have investigated the relationships among psychological capital, coping style, and prenatal depression in pregnant women. Therefore, exploring the mechanism and path analysis of these factors is important for improving the mental health of pregnant women during pregnancy. The conceptual framework of this study is primarily grounded in the Conservation of Resources Theory [19] and Lazarus′ Transactional Theory of Stress and Coping [20]. According to these theoretical perspectives, pregnancy can be viewed as a process involving potential resource loss, whereas psychological capital (hope, resilience, optimism, and self‐efficacy) is regarded as a key personal resource. When facing stressful situations such as pregnancy, individuals engage in cognitive appraisal and adopt corresponding coping strategies. We hypothesize that pregnant women with abundant psychological resources can more effectively utilize positive coping strategies—a form of resource investment—to buffer against stress and prevent prenatal depression, which represents an outcome of resource depletion. Conversely, individuals with insufficient psychological capital may, owing to resource scarcity, tend to adopt negative coping styles, leading to further resource loss and thereby increasing the risk of depression. Therefore, we propose a mediation model to examine whether coping styles serve as the intrinsic mechanism through which psychological capital influences prenatal depression, thereby providing a theoretical reference for psychological interventions targeting prenatal depression.

## 2. Methods

### 2.1. Study Design

This study employed a quantitative, cross‐sectional research design. A convenience sample of pregnant women was recruited to complete a structured questionnaire at a single time point. The design was deemed appropriate for assessing the prevalence of prenatal depression and examining the relationships between key variables (prenatal depression, psychological capital, and coping styles) at a specific moment in time. The Strengthening the Reporting of Observational Studies in Epidemiology (STROBE) guidelines for cross‐sectional studies were consulted to ensure comprehensive reporting [21] (Supporting information [available here]).

### 2.2. Participants

This was a cross‐sectional survey conducted in the obstetric outpatient department of two hospitals in Guangzhou and Zhongshan, Guangdong Province, from March 2024 to December 2024. A questionnaire survey was distributed among pregnant women by means of a convenience sampling method. The inclusion criteria for the pregnant women were as follows: (a) gestational age ranging from 13 to 40 weeks and age > 20 years; (b) no family history of mental illness; and (c) the ability to understand and complete the questionnaire voluntarily. The exclusion criterion was serious physical illness leading to the inability to complete the questionnaire.

The following formula was used to calculate the sample size: $n={Z_{\alpha /2}}^{2}\times \left(P\times \left(1-P\right)\right)/\varepsilon^{2}$. According to the results of previous studies, the detection rate of prenatal depression is approximately 24.40% [2]. When the confidence level is 95%, *Z*_*α*/2_ = 1.96. Taking the error value (*ε*) = 5%, the sample size was expanded by 20% to avoid invalid questionnaires, resulting in 355 cases. This study included 769 pregnant women.

### 2.3. Measurements

The questionnaire used in this study included a general data questionnaire, the Edinburgh Postnatal Depression Scale (EPDS), the Simplified Coping Style Questionnaire (SCSQ), and the Positive PsyCap Questionnaire (PPQ). We used the Chinese version of these scales in this study.

#### 2.3.1. Sociodemographic Characteristics

The general data questionnaire used was prepared by the researcher and included the following: age, gestation, household registration, educational level, employment status, and parity.

#### 2.3.2. EPDS

The EPDS was developed by Cox et al. [22] in 1978 and revised by Lee, a researcher at the Chinese University of Hong Kong, in 1998 [23]. The scale consists of 10 items, namely, mood, fun, self‐blame, anxiety, fear, insomnia, ability to cope, sadness, crying, and self‐injury. The EPDS is scored on a 4‐point Likert scale (0–3 points). The scores of each item were summed to obtain the total score, which ranged from 0 to 30 points. A range of EPDS cutoff values (9, 10, 12, and 13) has been used in previous research. The 9/10 threshold is recommended by both Cox [22] and the Chinese version [23] for primary care screening. To align with our goal of optimizing sensitivity for the early identification of at‐risk pregnant women, a lower cutoff of 9 was selected, with higher scores indicating higher levels of depression. In this study, the Cronbach’s *α* coefficient of this scale was 0.885.

#### 2.3.3. SCSQ

The SCSQ was developed by the Chinese scholar Xie [24] in 1998. The scale consists of 20 items, including two dimensions, namely, positive coping and negative coping, which are used to reflect attitudes and approaches to daily living. The SCSQ is scored on a 4‐point Likert scale (0–3). The positive coping dimension score is equal to the average score of the sum of all positive items, and the negative coping dimension score is equal to the average score of the sum of all negative items. The higher the score is, the greater the likelihood that a response will be used. In this study, the Cronbach’s *α* coefficients of the positive and negative coping dimensions were 0.863 and 0.796, respectively, and the total Cronbach’s *α* was 0.845.

#### 2.3.4. PPQ

This study used the positive psychological capital questionnaire (PPQ) developed by Zhang et al. [25]. There are 26 items in the PPQ that are divided into four dimensions: self‐efficacy, resilience, hope, and optimism. The PPQ is scored on a 7‐point Likert scale (1–7 points), and the total score ranges between 26 and 182 points. A higher score corresponds to a higher level of psychological capital and a more positive individual. In this study, the Cronbach’s *α* coefficients of self‐efficacy, resilience, hope, and optimism were 0.787, 0.721, 0.837, and 0.858, respectively, and the total Cronbach’s *α* coefficient was 0.925.

### 2.4. Data Collection

We conducted a survey in the obstetric outpatient department of two hospitals in Guangzhou and Zhongshan, Guangdong Province, from March 2024 to December 2024. Before the study began, our researchers received special training, including instructions regarding the questionnaires and the purpose of the survey. The researchers first explained the purpose, process, and precautions of the study to pregnant women and their families, and all their information was kept strictly confidential. After informed consent was obtained from the patient, the patient received the questionnaires. After the questionnaire was returned, the researcher checked whether the questionnaire was completed and corrected any leakage or filling errors in time. Throughout the process, we respected the right of each pregnant woman to be informed, voluntarily consent to the study, or withdraw from the study, and we encouraged and helped each pregnant woman with *t* scores greater than 9 on the EPDS or with suicidal thoughts to seek psychological counseling and treatment.

### 2.5. Statistical Analysis

The data were entered into EpiData software and analyzed with SPSS 25.0 software. Count data are presented as frequencies and percentages, and continuous data are presented as the means ± standard deviations (M ± SD). Group differences in baseline characteristics were assessed using the *χ* ^2^ test for categorical variables and the independent samples *t*‐test for normally distributed continuous variables. For continuous data that did not meet the assumption of normality, the Mann‒Whitney *U* test was employed. Spearman correlation analysis was applied to explore the correlations among coping styles, psychological capital, and prenatal depression. Mplus 8.3 software was used to construct a mediation model between psychological capital and prenatal depression. All the significance tests were two‐sided, with a significance level of 0.05.

The model fitting test indices included the following: (1) Chi‐square test: Its significance suggests whether to accept the hypothetical model proposed by the study. Moreover, this indicator is very sensitive to sample size and tends to increase with increasing sample size. Generally, a *χ* ^2^ */df* less than 5 indicates that the model is well fitted. (2) Toot mean square error of approximation (RMSEA): An RMSEA less than 0.08 is considered an acceptable model, and an RMSEA less than 0.05 is considered the threshold of a good model. (3) Comparison of the fit index (CFI) and the noncanonical fit index (NNFI, also known as the Tucker‒Lewis index, TLI): A CFI and TLI greater than 0.90 indicates good model fit. (4) Standardized residual root mean square (SRMR): The SRMR is a fitting index used to directly evaluate the residuals. The values range from 0 to 1, and when the value is less than 0.08, the model is reasonable. (5) The Akaike information criterion (AIC) and the Bayesian information criterion (BIC): The AIC and BIC were used to compare nonnested models. The smaller these values are, the better the model fit is.

## 3. Results

### 3.1. Participant Characteristics

The 769 pregnant women ranged in age from 23 to 45 years, with a mean age of 31.02 ± 3.45 years. Among these pregnant women, the gestational age ranged from 13 to 40 weeks, with a mean of 30.01 ± 6.74 weeks. The majority of participants (63.20%) were primiparous. Most of the women (93.37%) had rural household registration, most (69.05%) had a university degree or above, and 81.40% had full‐time jobs (see Table 1).

**TABLE 1:** General data and scale assessment scores of pregnant women.

	**Total**	**Depressed**	**Nondepressed**	**χ^2^/*Z***	**p**

Household registration					
Town	51 (6.63%)	23 (45.10%)	28 (54.90%)	2.56^a^	0.11
Country	718 (93.37%)	244 (33.98%)	474 (66.02%)		
Level of education					
Bachelor’s degree or above	531 (69.05%)	181 (34.09%)	350 (65.91%)	0.42^a^	0.81
Junior college	199 (25.88%)	71 (35.68%)	128 (64.32%)		
High school and below	39 (5.07%)	15 (38.46%)	24 (61.54%)		
Employment status					
Full‐time	626 (81.40%)	209 (33.39%)	417 (66.61%)	3.12^a^	0.21
Part‐time	79 (10.27%)	34 (43.04%)	45 (56.96%)		
Unemployed	64 (8.32%)	24 (37.5%)	40 (62.50%)		
Parity					
Primipara	486 (63.20%)	172 (35.39)	314 (64.61%)	0.26^a^	0.61
Multipara	283 (36.80%)	95 (33.57%)	188 (66.43%)		
Age (years)	31.02 ± 3.45	30.86 ± 3.28	31.11 ± 3.53	−0.77^b^	0.44
Gestation (weeks)	30.01 ± 6.74	29.79 ± 6.81	30.13 ± 6.71	−0.60^b^	0.55
EPDS	7.71 ± 5.07	13.32 ± 3.37	4.73 ± 2.78	37.82^b^	< 0.001
Positive coping	2.04 ± 0.50	1.89 ± 0.48	2.11 ± 0.50	−6.04^b^	< 0.001
Negative coping	1.15 ± 0.54	1.25 ± 0.53	1.10 ± 0.54	3.75^b^	< 0.001
Self‐efficacy	31.51 ± 6.04	28.90 ± 5.72	32.91 ± 5.75	−9.23^b^	< 0.001
Resilience	30.42 ± 5.44	27.54 ± 4.48	31.94 ± 5.30	−11.56^b^	< 0.001
Hope	29.93 ± 5.20	27.88 ± 4.92	31.02 ± 5.01	−8.34^b^	< 0.001
Optimism	30.04 ± 5.20	27.64 ± 5.16	31.33 ± 4.76	−9.94^b^	< 0.001
PPQ	121.91 ± 18.78	111.96 ± 17.00	127.20 ± 17.50	−11.61^b^	< 0.001

Abbreviations: EPDS, Edinburgh Postnatal Depression Scale; PPQ, Positive PsyCap Questionnaire.

^a^ *χ* ^2^ test.

^b^Mann‒Whitney *U* test.

### 3.2. Psychological Capital, Coping Style, and Prenatal Depression Score

The total scores for psychological capital, positive coping, negative coping, and prenatal depression for the 769 pregnant women were 121.91 ± 18.78, 2.04 ± 0.50, 1.15 ± 0.54, and 7.71 ± 5.07, respectively. In total, 34.7% (269) of the pregnant women were tested for prenatal depression. There was no significant difference in general demographic data (e.g., age, gestation, household registration, educational level, employment status, and parity) between depressed pregnant women and nondepressed pregnant women (all *p* > 0.05). However, there were significant differences between the two groups in terms of the EPDS total score, positive coping, negative coping, psychological capital, and total psychological capital (all *p* < 0.001). Specifically, pregnant women in the nondepressed group tended to adopt positive coping styles, the scores for psychological capital and its dimensions were higher in the nondepressed group than in the depressed group, and the psychological capital level of the nondepressed group was higher. The scores of each scale and dimension are shown in Table 1.

### 3.3. Spearman Correlation Analysis of Prenatal Depression With Psychological Capital and Coping Style

After controlling for general demographic data (e.g., age, gestation, household registration, educational level, employment status, and parity), spearman correlation analysis revealed that psychological capital and its dimensions (self‐efficacy, resilience, hope, and optimism) were positively correlated with positive coping (*r* = 0.44, 0.35, 0.36, 0.42, and 0.38, all *p* < 0.001) and negatively correlated with prenatal depression (*r* = −0.46, −0.38, −0.45, −0.34, and −0.41, all *p* < 0.001) (Table 2). Moreover, this analysis indicated that positive coping was negatively correlated with prenatal depression (*r* = −0.26, *p* < 0.001) and that negative coping was positively correlated with prenatal depression (*r* = 0.23, *p* < 0.01) (Table 2).

**TABLE 2:** Spearman correlation analysis of prenatal depression, psychological capital, and coping style in pregnant women (*n* = 769, *r*).

	**EPDS**	**Positive coping**	**Negative coping**	**PPQ**	**Self-efficacy**	**Resilience**	**Hope**	**Optimism**

EPDS	1.00	−0.26^∗∗^	0.18^∗∗^	−0.46^∗∗^	−0.38^∗∗^	−0.45^∗∗^	−0.34^∗∗^	−0.41^∗∗^
Positive coping	−0.26^∗∗^	1.00	0.23^∗∗^	0.44^∗∗^	0.35^∗∗^	0.36^∗∗^	0.42^∗∗^	0.38^∗∗^
Negative coping	0.18^∗∗^	0.23^∗∗^	1.00	−0.14^∗∗^	−0.13^∗∗^	−0.15^∗∗^	−0.15^∗∗^	−0.04
PPQ	−0.46^∗∗^	0.44^∗∗^	−0.14^∗∗^	1.00	0.86^∗∗^	0.81^∗∗^	0.87^∗∗^	0.87^∗∗^
Self‐efficacy	−0.38^∗∗^	0.35^∗∗^	−0.13^∗∗^	0.86^∗∗^	1.00^∗∗^	0.68^∗∗^	0.67^∗∗^	0.66^∗∗^
Resilience	−0.45^∗∗^	0.36^∗∗^	−0.15^∗∗^	0.81^∗∗^	0.68^∗∗^	1.00	0.52^∗∗^	0.54^∗∗^
Hope	−0.34^∗∗^	0.42^∗∗^	−0.15^∗∗^	0.87^∗∗^	0.67^∗∗^	0.52^∗∗^	1.00	0.81^∗∗^
Optimism	−0.41^∗∗^	0.38^∗∗^	−0.04	0.87^∗∗^	0.66^∗∗^	0.54^∗∗^	0.81^∗∗^	1.00

Abbreviations: EPDS, Edinburgh Postnatal Depression Scale; PPQ, Positive PsyCap Questionnaire.

^∗∗^ *p* < 0.001.

### 3.4. Mediating Role of Coping Styles in the Relationship Between Psychological Capital and Prenatal Depression in Pregnant Women

In this study, psychological capital was used as the independent variable, the four dimensions of psychological capital (self‐efficacy, resilience, hope, and optimism) were used as the latent variables, the two dimensions of coping style (positive coping and negative coping) were used as the mediating variables, and prenatal depression was used as the dependent variable to construct a research hypothesis model. The bootstrap sampling method (5000 samples) was used to verify whether coping style played a mediating role between psychological capital and prenatal depression. After the initial model was run, the fit of all the indicators was satisfactory, but the degree of fit was slightly poor, with *TLI* = 0.76 < 0.900 and *RMSEA* = 0.190 > 0.100. Therefore, it was necessary to revise the model. The fit indices of the modified model were *CFI* = 0.98, *TLI* = 0.94, *RMSEA* = 0.09, *SRMR* = 0.03, and *χ* ^2^ */df* = 7.63. Given that the majority of indices (CFI, TLI, and SRMR) met the thresholds for good fit and that the RMSEA value was within the acceptable range, the model was deemed to have an acceptable overall fit to the data.

**FIGURE 1:**
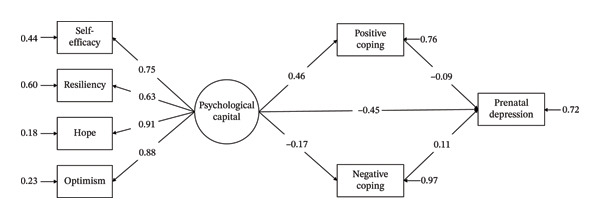
Standardized mediation model of psychological capital on prenatal depression.

The results of the mediation model revealed a significant direct effect of psychological capital on prenatal depression (*β* = −0.45, *p* < 0.001). Furthermore, psychological capital was significantly associated with both positive coping (*β* = 0.46, *p* < 0.001) and negative coping (*β* = −0.17, *p* < 0.001). Moreover, psychological capital decreased prenatal depression by increasing positive coping (*β* = −0.04; *p* < 0.001) and inhibiting negative coping (*β* = −0.02; *P* *p* < 0.001), and there was no significant difference in the mediating effect between positive and negative coping variables (*p* > 0.05). The estimated indirect effect of coping style was −0.06 (*p* < 0.001), and the mediating effect was −0.51 (*p* < 0.001), accounting for 11.87% of the total effect (*p* < 0.001). The interactions between variables are shown in Table 3 and Figure 1.

**TABLE 3:** Mediating role of coping styles in the relationship between psychological capital and prenatal depression.

Path	*β*	95% CI
Psychological capital ⟶ positive coping	0.46	0.04, 0.06
Psychological capital ⟶ negative coping	−0.17	−0.03, −0.01
Psychological capital ⟶ positive coping ⟶ prenatal depression	−0.04	−0.08, −0.01
Psychological capital ⟶ negative coping ⟶ prenatal depression	−0.02	−0.04, −0.01
Direct effect	−0.45	−0.54, −0.36
Total indirect effect	−0.06	−0.11, −0.02
Total mediation effect	−0.51	−0.58, −0.45

## 4. Discussion

In this study, the relationships among psychological capital, coping style, and prenatal depression were explored in pregnant women, and the following conclusions were drawn: Psychological capital has direct and indirect effects on prenatal depression, with the latter being realized through coping styles. The findings of this study highlight the important factors affecting prenatal depression. This information is helpful for exploring the complex mechanisms underlying prenatal depression and for increasing the attention given by nurses to the positive psychological capital of pregnant women.

### 4.1. Current Situation of Prenatal Depression in Pregnant Women

The mental health of pregnant women is a significant public health concern. The detection rate of prenatal depression in this study was 34.7%, which is substantially higher than the rates reported in studies from Tianjin (12.4% [26]) and Shenzhen (4.4% [27]). This discrepancy can be attributed primarily to two key methodological factors. First, and most importantly, the screening instruments differed. Our study used the EPDS, which is specifically designed and validated for the perinatal period, whereas the other studies used the SDS [26], which is a general depression scale that may lack sensitivity in pregnant populations. Second, the choice of cutoff score significantly influences prevalence estimates. We adopted the recommended cutoff of ≥ 9 to maximize sensitivity for early identification in a primary care context [22, 23], thereby including cases of mild severity. In contrast, the use of a higher cutoff (e.g., ≥ 13) targets more severe cases and yields lower rates [27, 28].

Beyond methodological differences, regional variations in social support systems, economic stressors, and cultural factors between Guangdong and other regions may also contribute to the observed differences [26, 27]. Furthermore, assessments during different trimesters of pregnancy could affect the rates. It is also important to note that despite the potential underreporting due to stigma associated with mental illness [29], our findings still revealed a high prevalence, underscoring the substantial burden of prenatal depression. The lack of significant associations with common sociodemographic variables, in contrast to the findings of some previous studies, may be due to the aforementioned methodological approaches or our single‐center sampling strategy [30], which limits generalizability. Nevertheless, the high detection rate observed under a sensitive screening protocol highlights prenatal depression as a prevalent and serious concern in this population. This motivates further investigation into region‐specific psychosocial determinants and underlying mechanisms to inform the development of targeted coping strategies.

### 4.2. Psychological Capital Can Directly Affect Prenatal Depression in Pregnant Women

This study revealed that higher levels of psychological capital and its dimensions (self‐efficacy, resilience, hope, and optimism) in pregnant women were significantly associated with a lower risk of prenatal depression. These findings align with the theoretical framework proposed at the beginning of this study, which posits that positive emotional and cognitive resources can counteract the impact of negative stressors [31, 32]. As an integrated positive resource, psychological capital equips individuals with the capacity to proactively manage challenges and maintain psychological well‐being during stressful life transitions such as pregnancy [33]. The same conclusions have been reached in previous studies [34].

The significant negative correlation observed between psychological capital and prenatal depression can be understood by examining the unique role of each dimension. Self‐efficacy reflects an individual’s confidence in mobilizing cognitive resources and executing actions to cope with demands [35], which may help explain its regulatory effect on depressive symptoms, as reported by Wang et al. [36]. Resilience, the ability to recover from adversity, allows pregnant women to adapt more effectively to physical and emotional changes. Moreover, hope and optimism foster a positive outlook toward the future, sustaining motivation and reducing anxiety [32, 37]. Our results are consistent with the work of Tuxunjiang et al. [38], who also highlighted the protective role of positive psychological resources against negative emotions in pregnant women. This convergence of findings underscores the importance of viewing psychological capital as a multifaceted construct that collectively contributes to mental health.

On the basis of these findings, future interventions for pregnant women should move beyond a singular focus and aim to holistically enhance all dimensions of psychological capital, thereby increasing individuals′ reserves of positive psychological resources. Healthcare providers could help strengthen self‐efficacy through mastery experiences and skill‐building, foster resilience using stress‐management techniques, and cultivate hope and optimism through future‐oriented goal‐setting and cognitive restructuring. Such a comprehensive, multidimensional strategy is more likely to effectively alleviate symptoms of prenatal depression and promote overall psychological well‐being in pregnant women.

### 4.3. Pregnant Women’s Psychological Capital Can Indirectly Affect Antenatal Depression Through Coping Styles

More importantly, we found that coping styles serve as a significant intermediary pathway in the relationship between psychological capital and prenatal depression. This mediation effect operates in a dual manner: higher psychological capital predicts both increased use of positive coping styles and decreased reliance on negative coping styles. These adaptive coping responses are associated with lower levels of depressive symptoms. This statistically indirect effect suggests that fostering psychological capital may help reduce prenatal depression not only directly but also by promoting more adaptive stress management strategies.

This finding is well embedded within existing theoretical and empirical work. It aligns with the broader notion that personal resources influence mental health outcomes through behavioral mechanisms. For example, our results resonate with studies indicating that resilience and self‐efficacy are linked to more positive and less negative coping, which protects against depression [6, 18, 39]. Similarly, research on other populations has shown that individuals high in hope and optimism typically employ more proactive coping strategies to mitigate distress [40], whereas a reliance on negative, avoidant coping exacerbates psychological pressure [41]. The consistency between our model and these prior findings (e.g., [42]) strengthens the plausibility of the proposed mediation pathway.

Pregnancy is a unique period of life because of the variety of associated physical, social, and psychological factors, and these factors can place a certain amount of pressure on women and lead to anxiety, depression, and other emotional problems [29]. Given this context, interventions that empower women to manage such pressures are especially valuable. Specifically, identifying ways to help pregnant women tap into their own psychological resources—such as those encompassed by psychological capital—to mitigate depression is of particular importance.

In line with this need, our results suggest that programs aimed at preventing prenatal depression could yield greater benefits by simultaneously enhancing psychological capital (e.g., by increasing self‐efficacy, resilience, hope, and optimism) and directly coaching pregnant women regarding effective coping skills. Helping women develop these interrelated resources may provide a more robust buffer against the unique stresses of pregnancy.

### 4.4. Strengths and Limitations

Our study has several strengths. First, the sample size of the study subjects was sufficient to test the mediation effect. Second, the conceptual framework, which integrates the Conservation of Resources Theory and Lazarus’ Transactional Theory of Stress and Coping, provides a reference to help specialists understand the potential mechanism through which coping styles affect prenatal depression in pregnant women and how psychological capital affects prenatal depression. However, several limitations should be noted. First, our study participants were recruited from only two hospitals in Guangdong Province, China, which may limit the generalizability of the findings to other regions or healthcare settings. In addition, the cross‐sectional nature of the study precludes definitive conclusions regarding the causal direction of the observed relationships. Although our model posits that psychological capital influences coping strategies, which in turn affect depressive symptoms, alternative interpretations remain statistically plausible. Therefore, these results suggest a potential mechanism that requires further validation through longitudinal or experimental studies.

## 5. Conclusion

Psychological capital has both direct and indirect effects on prenatal depression, with the indirect pathway operating through the mediation of coping styles—by fostering positive coping strategies and reducing reliance on negative ones. These findings suggest that healthcare providers should incorporate psychological capital assessment into routine prenatal care for pregnant women and develop interventions aimed at enhancing positive psychological resources while promoting adaptive coping strategies, thereby effectively mitigating the risk of prenatal depression.

## 6. Relevance to Clinical Practice

Since psychological capital indirectly influences depression through coping strategies, managing prenatal depression requires a dual approach. First, the positive coping mechanisms of pregnant women can be enhanced. Through targeted interventions, caregivers can promote positive psychological interventions to systematically improve pregnant women’s self‐efficacy, resilience, hope, and optimism. For example, activities that build self‐efficacy and encourage proactive coping—such as structured problem‐solving exercises, goal‐setting in daily routines, or guided mindfulness‐based stress reduction—can help pregnant women strengthen their sense of control and reduce reliance on passive or avoidant responses.

Second, it is important to help pregnant women identify avoidance behaviors or emotional suppression tendencies. Techniques such as cognitive behavioral therapy can be used to help pregnant women identify and improve negative coping patterns. For example, a coping style diary—where pregnant women document daily stressors and their responses—allows caregivers to provide tailored feedback and improvement strategies. Healthcare institutions should establish multidisciplinary collaboration mechanisms that integrate resources from obstetrics, mental health departments, and community services to construct a stepped‐care intervention model. The theoretical framework integrated in this study provides practical guidance for preventing prenatal depression: an intervention approach focused on enhancing psychological resources as the foundation and improving coping strategies as the pathway. This framework can not only effectively prevent prenatal depression but also promote pregnant women’s physical and mental health through empowerment rather than correction, ultimately improving mother and infant health outcomes.

## Author Contributions

Study​ design: Wenting Liu, Yibing Tan, Xiaowan Luo, and Lili Deng.

Data collection, analysis, and interpretation: Weiting Li, Min Li, Honglin Chen, Yifei Wang, Jiagen Xiang, and Ming Yang.

Drafting of the manuscript: Wenting Liu.

Critical revision of the manuscript: Wenting Liu and Lili Deng.

Approval of the final version for publication: all coauthors.

## Funding

This research was supported by the Humanities and Social Sciences Research Planning Fund Project of the Ministry of Education (grant number: 25YJA630097).

## Ethics Statement

Ethical approval was obtained (ref no. KY202200401), and participation in the study was voluntary. Participants were informed about the research on the planned data collection date, and their informed consent was obtained.

## Consent

The authors have nothing to report.

## Conflicts of Interest

The authors declare no conflicts of interest.

## Supporting Information

Additional supporting information can be found online in the Supporting Information section.

## Supporting information


**Supporting Information** The STROBE checklist cross‐sectional to this article can be found online.

## Data Availability

The dataset supporting the conclusions of this article is included within the article.
